# Supplementary figures and images for: Single-cell RNA sequencing of nc886, a non-coding RNA transcribed by RNA polymerase III, with a primer spike-in strategy
Source: PLoS One. 2024 Aug 27;19(8):e0301562. doi: 10.1371/journal.pone.0301562 (PMC11349216; doi:10.1371/journal.pone.0301562)

A.

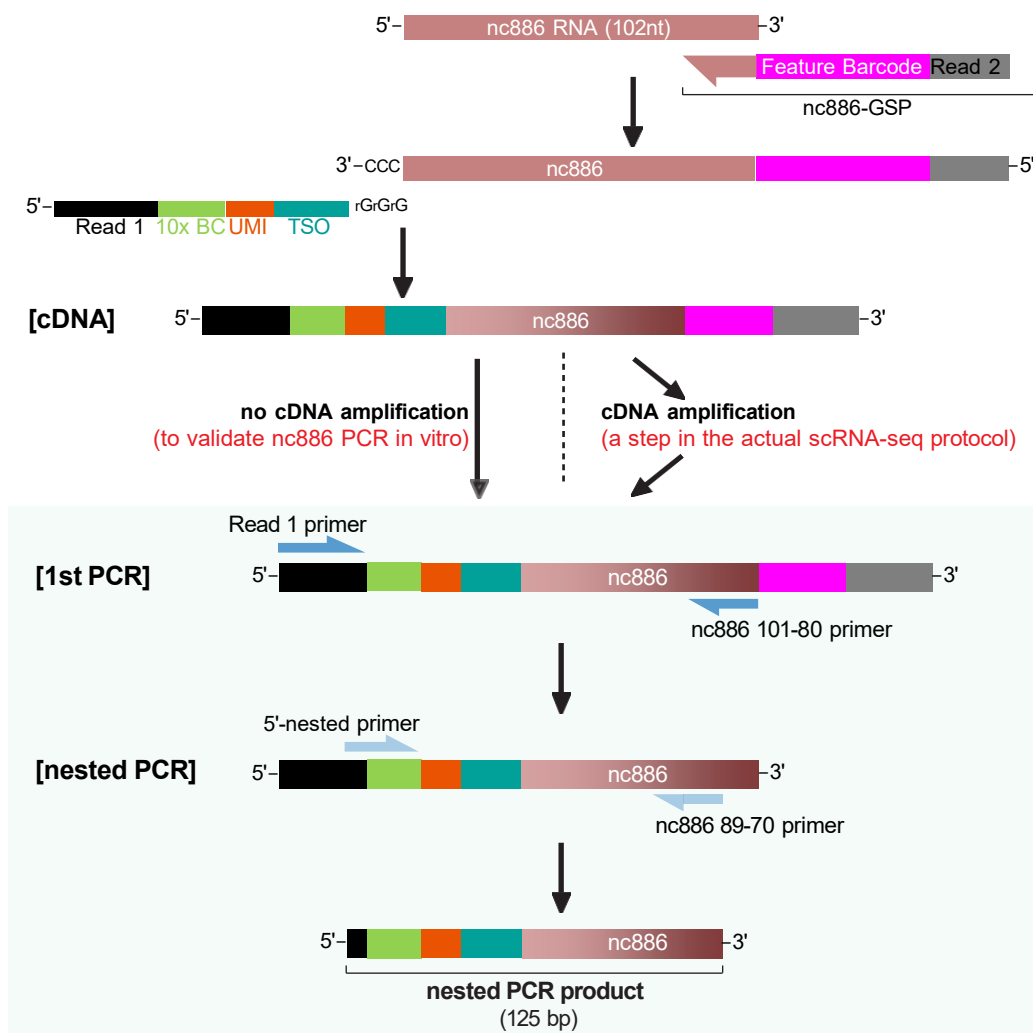

B.

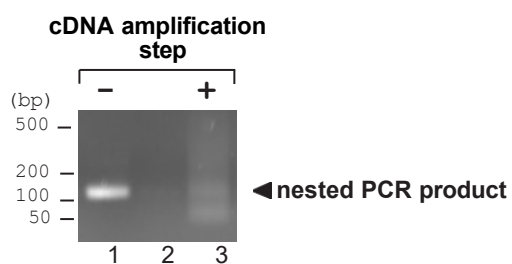

C.

melting curves of the nested PCR products

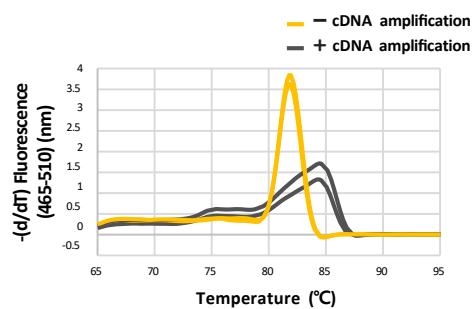

Supplement: S1 Fig — (A) Schematic diagram of recapitulation of the scRNA-seq steps in vitro. Total RNA isolated from Huh7, a hepatoma cell line expressing nc886 abundantly, was subjected to cDNA synthesis and PCR amplification, with or without the cDNA amplification step. Primer sequences (5’ to 3’) are: Read 1, ctacacgacgctcttccgatct; nc886 101–80, aagggtcagtaagcacccgcg; 5’-nested, ccgatctaaacctgagaaacc; nc886 89–70, ggtctcgaaccccagcacag. (B-C) 2% agarose gel electrophoresis and ethidium bromide staining visualization (panel B) and melting curves (panel C) of the nest PCR product. The 1st PCR was performed with LightCycler 480 SYBR Green I MasterMix (Roche, Penzberg, Germany), with cDNA from 20 ng of total RNA. The nested PCR was done with LightCycler 480 SYBR Green I MasterMix (Roche, Penzberg, Germany), using 1/200 (the + cDNA amplification sample) or 1/1000 (the—cDNA amplification sample) of the 1st PCR product. In panel B, the molecular sizes of 100bp Opti-DNA Marker (Applied Biological Materials; Richmond, Canada) are shown on the left. Lane 2 is blank. (PDF) [file pone.0301562.s001.pdf]

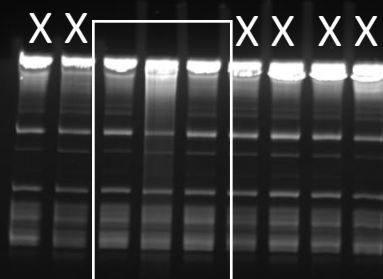

Used in Figure 1A EtBr staining

9/13  
cell lines nc886 level  
n days

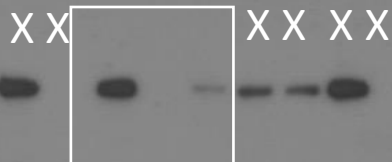

Used in Figure 1A nc886 Northern blot

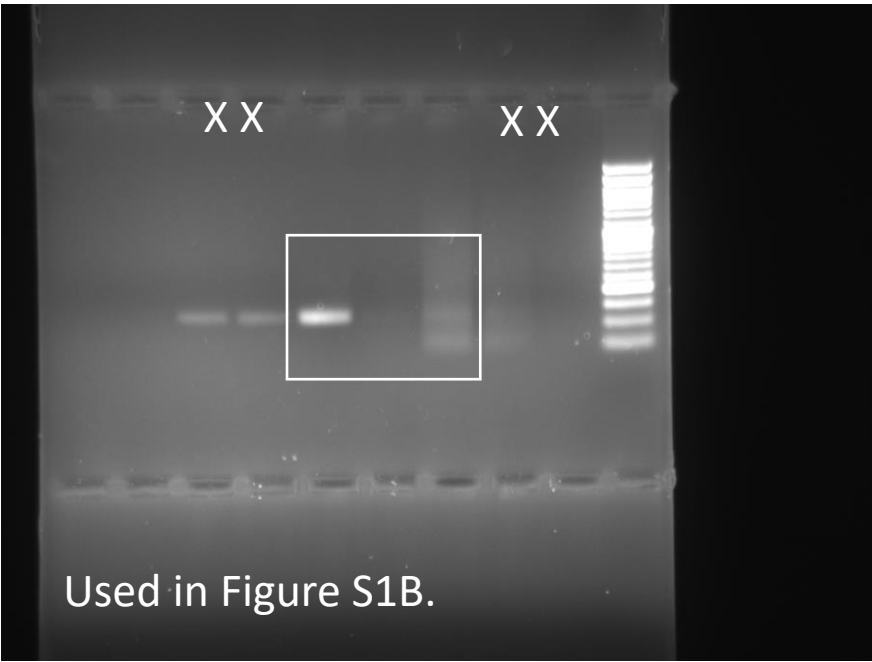

Supplement: S1 Raw images — (PDF) [file pone.0301562.s002.pdf]
