# Supplementary material for: Single-cell RNA sequencing of nc886, a non-coding RNA transcribed by RNA polymerase III, with a primer spike-in strategy
Source: PLoS One. 2024 Aug 27;19(8):e0301562. doi: 10.1371/journal.pone.0301562 (PMC11349216; doi:10.1371/journal.pone.0301562)
Supplement: Attachment — Submitted filename: Dear Editor.docx [file pone.0301562.s003.docx]

Dear Editor

I have questions regarding the experimental design that require clarification from the authors?

1.Limited ncRNA Coverage: While the developed methodology represents a significant step in addressing the challenge of profiling non-coding RNAs (ncRNAs) transcribed by RNA polymerase III (Pol III), it may still have limitations in capturing all types of ncRNAs. It's possible that other classes of ncRNAs or specific transcripts may not be effectively captured using this approach. How do the authors plan to address these potential limitations?

2. Oligo Tag Specificity: The authors note that the identification of oligo tags showed limited target specificity, implying that the oligo-tagged primer approach may not be completely specific to the target ncRNA. This raises concerns about potential off-target effects or noise in the sequencing data. Have the authors encountered off-target effects during their experimentation? How did they evaluate the specificity of the tags for other ncRNAs?

3. Validation and Reproducibility: While the methodology was applied to three different cell lines with varying levels of nc886 expression, further validation across a larger and more diverse set of cell types, tissues, or biological conditions would enhance the robustness and generalizability of the findings. Additionally, ensuring reproducibility of the results across different experimental setups and laboratories is essential. The authors' validation of this approach and the reproducibility of the results are important aspects to consider. How authors will address it?
